# Supplementary material for: Contrast-enhanced ultrasound with sub-micron sized contrast agents detects insulitis in mouse models of type1 diabetes
Source: Nat Commun. 2020 May 7;11:2238. doi: 10.1038/s41467-020-15957-8 (PMC7206014; doi:10.1038/s41467-020-15957-8)
Supplement: Supplementary file 1 — Supplementary Information [file 41467_2020_15957_MOESM1_ESM.pdf]

Supplementary Information

Contrast-enhanced ultrasound with sub-micron sized contrast agents detects insulitis in mouse models of type1 diabetes

Ramirez et al

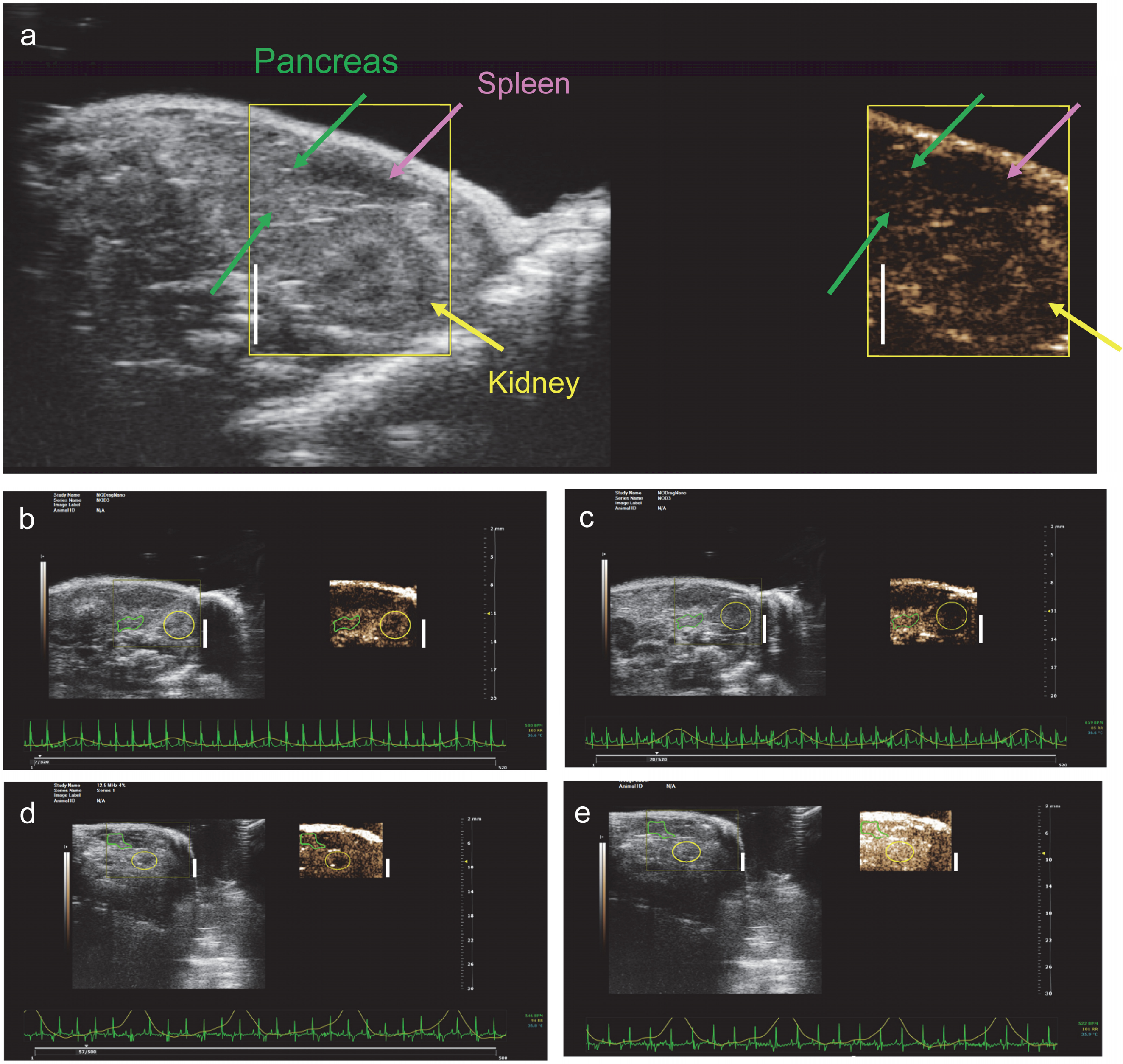

Supplementary Figure 1: Identification of the pancreas in relation to other organs in the abdomen. (A) Following ultrasound transducer placement, the abdomen and stomach was located in B-mode. Using micromanipulators, the transducer was moved along the transverse plane until the pancreas, kidney, and spleen were identifiable. The striated appearance of the pancreas was used to confirm transducer placement in B-mode and then Contrast mode was engaged. (B) Screen shot showing representative images in B-mode and contrast mode in a NOD mouse prior to NB infusion using 18MHz frequency and 10% transmission power. (C) as in B in NOD mouse 20min after NB infusion indicating pancreas-specific sustained increase in sub-harmonic contrast signal. (D) Screen shot showing representative images in B-mode and contrast mode in a NOD mouse prior to NB infusion using 12.5MHz frequency and 4% transmission power. (E) as in D in NOD mouse 1min after NB infusion indicating rapid increase in sub-harmonic contrast signal throughout abdomen. Scale bar represents 3mm.

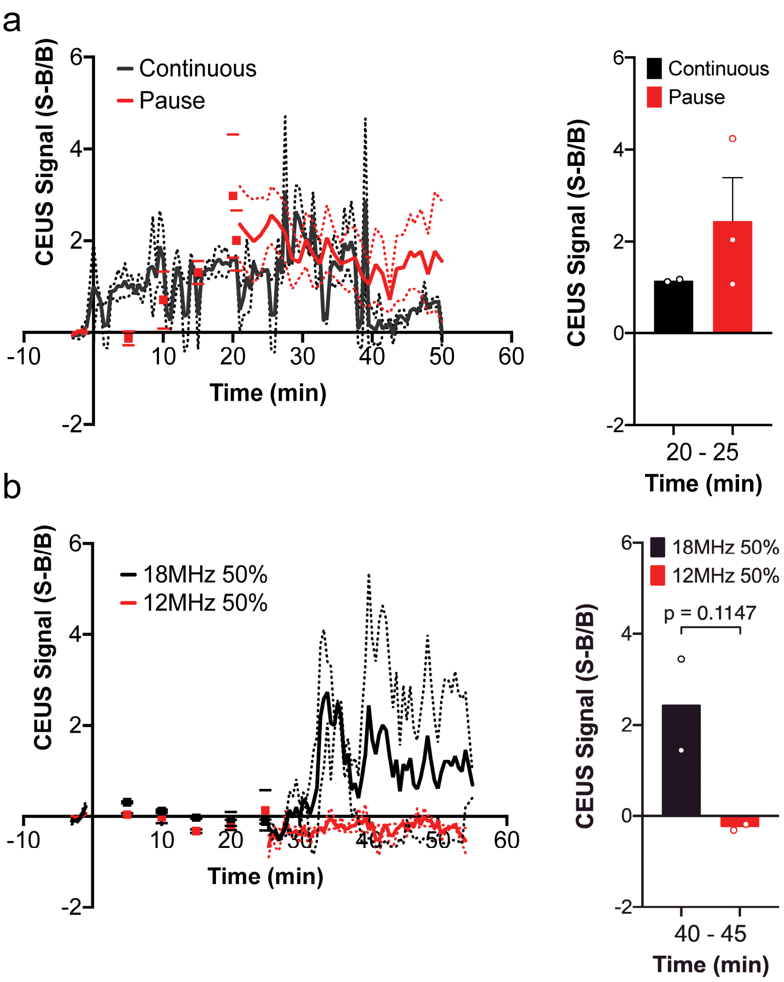

Supplementary Figure 2: Lack of impact of continual imaging on NB contrast signal. (A) Mean time-course of contrast signal in the pancreas of 10 week NOD mice, following either continuous imaging following NB infusion ('continuous'), as performed elsewhere in the study; or with only brief snap shot every 5min until 20min after NB delivery at which point continual imaging resumes ('pause') (left); together with the mean contrast signal averaged between 20-25 minutes (right). No significant difference in signal between 20 and 25 minutes was observed indicated (paired Student's t-test). (B) Mean time-course of contrast signal in the pancreas of 10 week NOD mice, using 18MHz or 12.5MHz center frequency, each at 50% transmission power, with brief snap shot every 5min until 20min after NB delivery at which point continual imaging resumes (left); together with the mean contrast signal averaged between 40-45 minutes (right). Error bars represent s.e.m. Data in A representative of n=2 or 3 mice, data in B representative of n=2 mice. Source data are provided as a Source Data file.

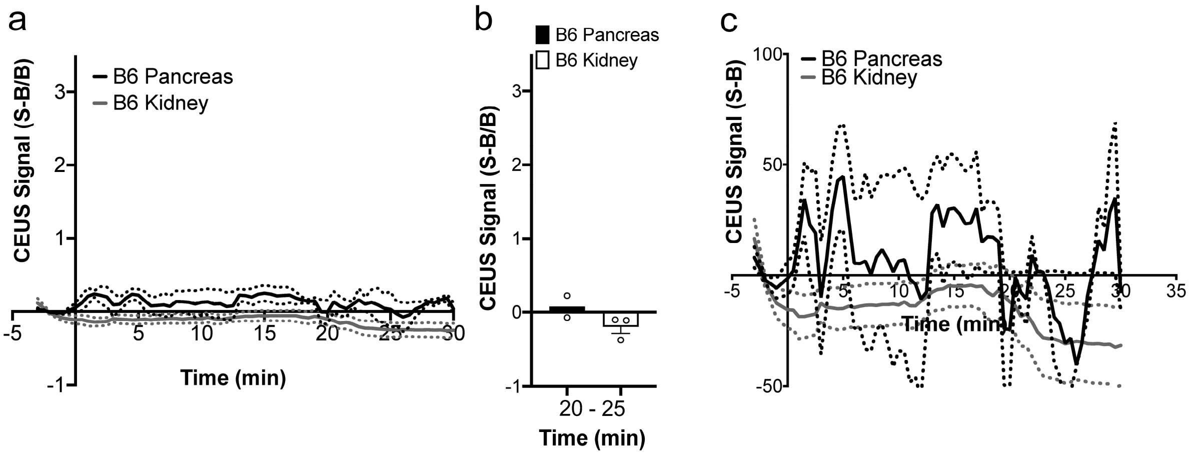

Supplementary Figure 3: NB time course and quantification of 10w C57BL/6J mice. Wildtype C57BL/6J mice were used to examine NB accumulation in a non-diseased model. (A) Mean time-course of contrast signal in the pancreas and kidney of 10 week old female C57BL/6J mice following NB infusion. (B) Mean contrast signal averaged between 20-25 minutes following NB infusion, in A. (C) As in A displaying the signal minus background (S-B). Data represents n=3 B6 mice. *$p<0.05$, comparing groups indicated (paired Student's t-test). Error bars in B represent s.e.m. Source data are provided as a Source Data file.

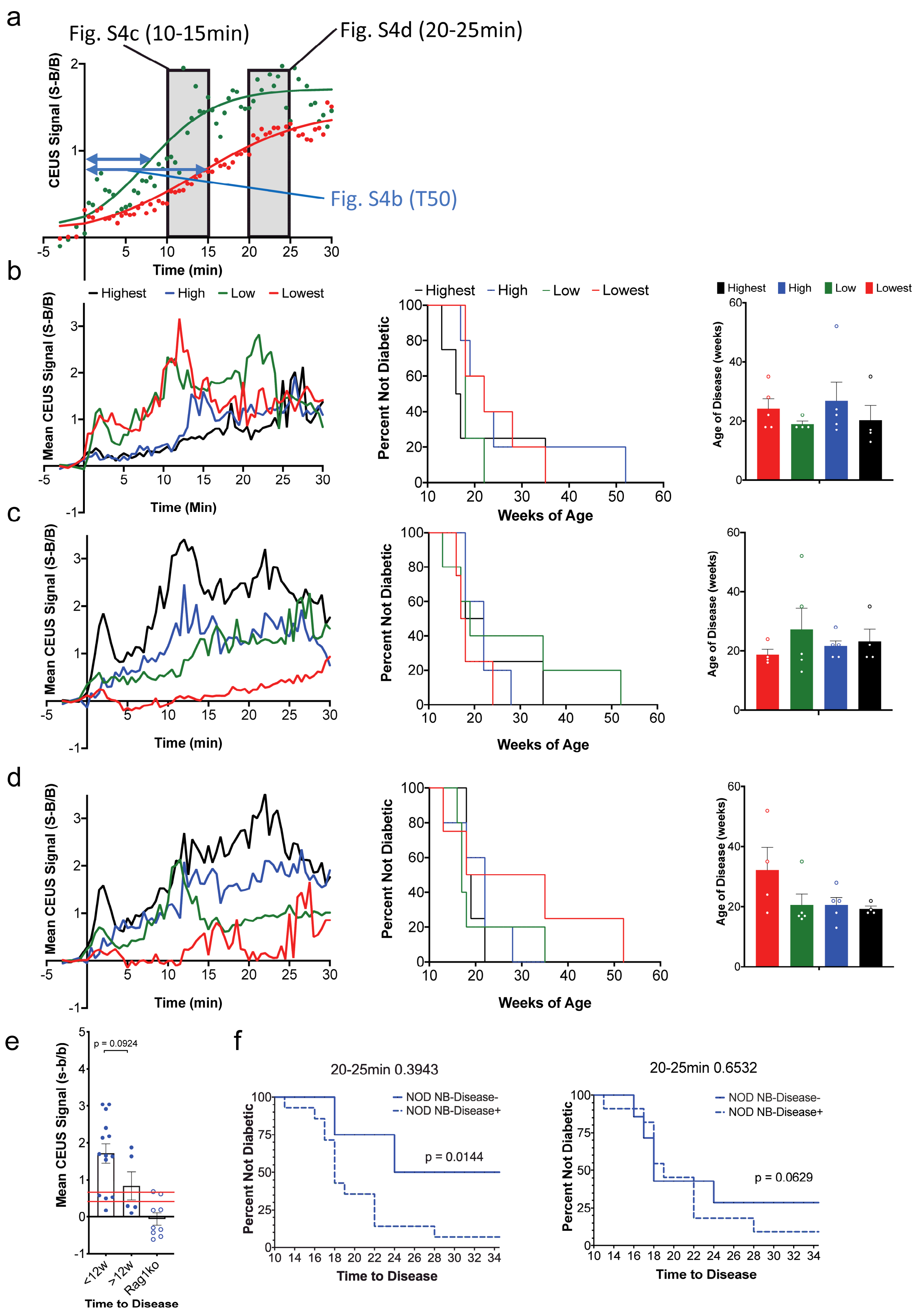

Supplementary Figure 4: Predicting diabetes progression. (A) Schematic of fitted time course indicating parameters we examined including time to 50% of maximal contrast elevation (t_{50}) and mean

contrast elevation between either 10-15 min. or 20-25 min. (B) Time-courses of contrast signal in the pancreas of 10 week old female NOD mice following NB infusion, grouped by quartiles according to the t_{50} (left), together with Kaplan Meier curves for each t_{50} quartile group (middle) and the age at which diabetes emerges for each t_{50} quartile group (right). (C) As in B with animals grouped by quartiles according to the mean contrast signal between 10-15 min. (D) As in B with animals grouped by quartiles according to the mean contrast signal between 20-25 min. (E) Mean contrast signal between 20-25 min. for those animals that develop diabetes at either less than 22 weeks age, or greater than 22 weeks age, together with corresponding Rag1ko data. Red lines indicate two threshold that separate NOD mice form Rag1ko mice. (F) Kaplan Meier curves for mice that show a mean contrast signal between 20-25 min. that is either greater than the threshold indicated in E (Disease +) or less than the threshold indicated in E (Disease -). Left panel indicates lower threshold and right panel indicates higher threshold. Error bars in B,C,D represent s.e.m. Data in B-E representative of n=18 mice. p value in E is calculated from paired Student's t-test, p value in F is calculated from a Mantel-Cox Logrank test. Source data are provided as a Source Data file.

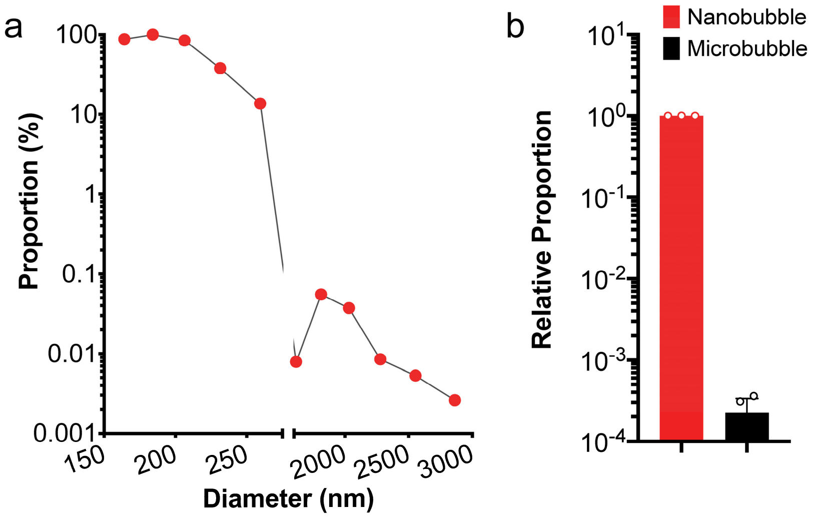

Supplementary Figure 5: Size distribution of NB sample. Following NB activation, the size distribution within the NB fraction was determined via resonant mass measurement (RMM) (Archimedes, Malvern Panalytical Inc., Westborough, MA, USA). (A) Median size distribution from n=3 sample measurements. (B) relative abundance of the nano-sized (<1μm) and micro-sized (>1μm). Error bars represent s.e.m. Third data point in B, microbubbles (relative proportion = 0) not displayed. Source data are provided as a Source Data file.

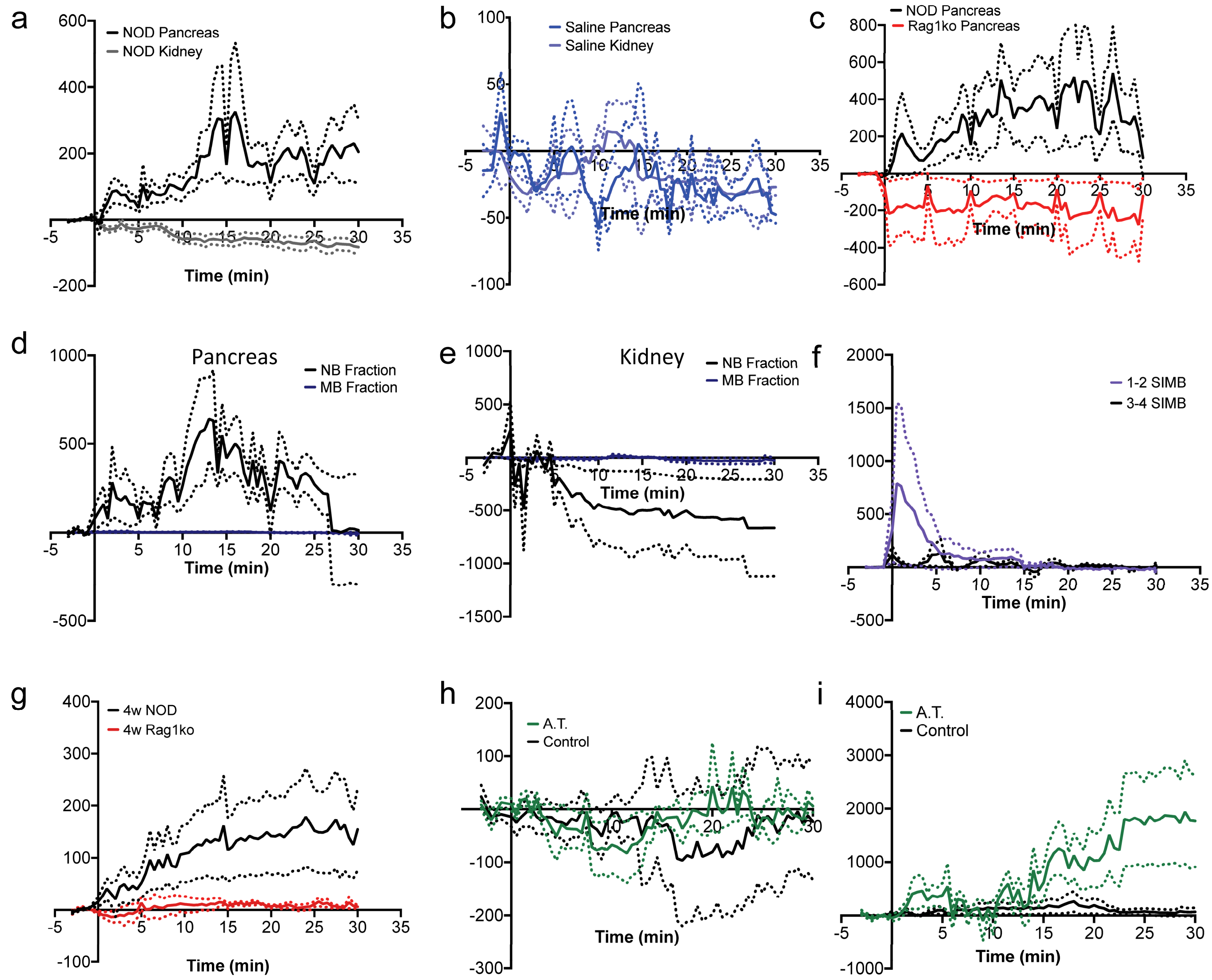

Supplementary Figure 6: NB time courses. Time courses displaying Signal minus Background (S-B) for those time-courses where S-B/B is displayed which includes an additional background normalization. (A) Data in Fig.1E. (B) Data in Fig.1F. (C) Data in Fig.1G. (D) Data in Fig.3E. (E) Data as in Fig.3E. showing for the kidney. (F) Data in Fig.3F. (G) Data in Fig.4A. (H) Data in Fig.5C. (I) Data as in Fig.5D showing for 4w following splenocyte infusion. Source data are provided as a Source Data file.
