# Supplementary material for: Contrast-enhanced ultrasound with sub-micron sized contrast agents detects insulitis in mouse models of type1 diabetes
Source: Nat Commun. 2020 May 7;11:2238. doi: 10.1038/s41467-020-15957-8 (PMC7206014; doi:10.1038/s41467-020-15957-8)
Supplement: Supplementary file 3 — Reporting Summary [file 41467_2020_15957_MOESM3_ESM.pdf]

nature research

Corresponding author(s): Richard KP Benninger

Last updated by author(s): 2/15/2020

Reporting Summary

Nature Research wishes to improve the reproducibility of the work that we publish. This form provides structure for consistency and transparency in reporting. For further information on Nature Research policies, see Authors & Referees and the Editorial Policy Checklist .

Statistics

For all statistical analyses, confirm that the following items are present in the figure legend, table legend, main text, or Methods section.

n/a	Confirmed	
[ ]	[x]	The exact sample size (*n*) for each experimental group/condition, given as a discrete number and unit of measurement
[ ]	[x]	A statement on whether measurements were taken from distinct samples or whether the same sample was measured repeatedly
[ ]	[x]	The statistical test(s) used AND whether they are one- or two-sided<br>*Only common tests should be described solely by name; describe more complex techniques in the Methods section.*
[x]	[ ]	A description of all covariates tested
[x]	[ ]	A description of any assumptions or corrections, such as tests of normality and adjustment for multiple comparisons
[ ]	[x]	A full description of the statistical parameters including central tendency (e.g. means) or other basic estimates (e.g. regression coefficient) AND variation (e.g. standard deviation) or associated estimates of uncertainty (e.g. confidence intervals)
[ ]	[x]	For null hypothesis testing, the test statistic (e.g. *F*, *t*, *r*) with confidence intervals, effect sizes, degrees of freedom and *P* value noted<br>*Give P values as exact values whenever suitable.*
[x]	[ ]	For Bayesian analysis, information on the choice of priors and Markov chain Monte Carlo settings
[x]	[ ]	For hierarchical and complex designs, identification of the appropriate level for tests and full reporting of outcomes
[x]	[ ]	Estimates of effect sizes (e.g. Cohen's *d*, Pearson's *r*), indicating how they were calculated

Our web collection on statistics for biologists contains articles on many of the points above.

Software and code

Policy information about availability of computer code

Data collection	Vevo Lab Version 1.7.0 (Build 7071); ZEN 2.3 blue edition (file version 2.3.69.01000)
Data analysis	VevoCQ Analysis; Matlab R2016b and R2018a; ImageJ Version: 2.0.0-rc-69/1.52i (Build: 269a0ad53f) - analyze particles macro.

For manuscripts utilizing custom algorithms or software that are central to the research but not yet described in published literature, software must be made available to editors/reviewers. We strongly encourage code deposition in a community repository (e.g. GitHub). See the Nature Research guidelines for submitting code & software for further information.

Data

Policy information about availability of data

All manuscripts must include a data availability statement. This statement should provide the following information, where applicable:
- Accession codes, unique identifiers, or web links for publicly available datasets
- A list of figures that have associated raw data
- A description of any restrictions on data availability

The data generated and analysed during the current study are available from the corresponding author on reasonable request. All raw imaging data associated with ultrasound, and histology is accessible from Zenodo.
Note, raw image and US data is too large to provide (100s GBs).We will assemble the raw data and upload this to Zenodo once we have requested permission to exceed their usual storage limits.

Field-specific reporting

Please select the one below that is the best fit for your research. If you are not sure, read the appropriate sections before making your selection.

Life sciences study design

All studies must disclose on these points even when the disclosure is negative.

Sample size	No formal sample size calculation was performed. 3 cohorts of 4 NOD mice per cohort were first examined as a minimum number of animals required to examine disease-dependent differences, given reported inter and intra cohort variability in disease development. This determination was based on significant variability in NOD mice disease progression in general, and specifically in the blood flow measured in NOD mice in prior studies (St Clair et al, Nat Comm 2018). In this study a difference was observed between pancreas and kidney after this initial minimum required number of animals.
Data exclusions	No data was excluded from analysis
Replication	2 different models of disease (NOD, adoptive transfer) were used, and 2 means to measure accumulation (ultrasound, histology) were performed. Each model and experimental approach reproduced initial fundings.
Randomization	Experimental animals were randomly assigned into relevant treatment groups.<br>where comparisons were made between different treatments (e.g. MB vs NB) measurements were made on the same animals, on separate days. (within 3 days). When caparison were made between animals, measurements were made on the same day. recordings were made in a randomized order
Blinding	Insulitis scoring was blinded. Contrast and fitting measurements were unbiased via automated matlab scripts (curve fitting or thresholding) and thus do not require blinding as analysis bias is negligible.

Reporting for specific materials, systems and methods

We require information from authors about some types of materials, experimental systems and methods used in many studies. Here, indicate whether each material, system or method listed is relevant to your study. If you are not sure if a list item applies to your research, read the appropriate section before selecting a response.

Materials & experimental systems

n/a	Involved in the study	
☐	☒	Antibodies
☒	☐	Eukaryotic cell lines
☒	☐	Palaeontology
☐	☒	Animals and other organisms
☒	☐	Human research participants
☒	☐	Clinical data

Methods

n/a	Involved in the study	
☒	☐	ChIP-seq
☒	☐	Flow cytometry
☒	☐	MRI-based neuroimaging

Antibodies

Antibodies used	guinea pig-anti-insulin primary (Autostainer Link 48, IR00261-2, Agilent/DAKO, Santa Clara, CA) diluted 1:5.<br>rabbit-anti-glucagon primary (2760S, Cell Signaling Technology, Danvers, MA) diluted 1:250.<br>AlexaFluor 555 goat anti-guinea pig secondary (A-21435, Thermo Fisher/Invitrogen, Carlsbad, CA) diluted 1:500.<br>AlexaFluor 488 donkey-anti-rabbit secondary antibody (A21206, Thermo Fisher/Invitrogen, Carlsbad, CA) diluted 1:500.<br><br>anti-mouse CD4 (BP0003-1; BioXCell). Note this was given for therapeutic purposes not for immunofluorescence or western blot analysis. Thus dilution is not applicable but treatment was a single 20 mg dose delivered IP
Validation	Insulin and glucagon antibodies validated in WT mice by lacking exocrine staining and showing specific staining in core and periphery of adult mouse islets.<br><br>Following anti mouse CD4 treatment, CD4+ T-cells were analyzed in peripheral blood (St Clair et al, Nat Comm 2018)

Animals and other organisms

Policy information about studies involving animals; ARRIVE guidelines recommended for reporting animal research

Laboratory animals	Mice: non-obese diabetic (NOD) female, 4w or 10w (JAX); NOD-scid female, 12-24w; C57bl/6 female, 8-16w; NOD-Rag1ko female 4w or<br>10w.
Wild animals	No wild animals were used

Field-collected samples	No filed collected samples were used
Ethics oversight	Institutional Animal Care and Use Committee of the University of Colorado Anschutz Medical campus B-95817(05)1D

Note that full information on the approval of the study protocol must also be provided in the manuscript.
